# Editorial Notes

**Published:** 1910-03

**Authors:** 


### Editorial Notes.

**The University of Bristol.**

Now that the University is a going concern, those prophets who foretold failure of the scheme may be reminded of the saying that "the impossible has become a fact by going through the hollow mockery of taking place." Not that those who worked for the scheme ever admitted that it was impossible, or that its "taking place" has been a hollow mockery. In a recent speech Sir Isambard Owen alluded to the prevailing misconception that when once the Charter was granted the University "had nothing to do but get up steam and go ahead." An immense amount of work has fallen on the members of all departments. For many months the Ordinances and Regulations have been under consideration, and very numerous meetings of the Boards of the various Faculties have been held. The Faculty of Medicine, in particular, has been very busy in constructing a curriculum of study for the approval of the Senate and Council. The course of study adopted by each of the newer Universities has been considered. The materials for the curriculum, as was the case in the celebrated "Deacon's Masterpiece," have been selected from those which experience seemed to show were best fitted for the requirements of the case. The aim has been to omit nothing essential, yet not to put an impossible burden on the student. It cannot, however, be expected or even hoped that, like the "wonderful one-hoss shay," the medical curriculum will survive unchanged until its hundredth anniversary.

Great difficulty has been experienced in selecting and adapting so as to prevent the curriculum becoming too cumbersome a machine. At first glance it might appear that the curriculum was intended to eliminate the weak by the process of survival of the fittest, whether teacher or student, and it has even grimly been compared to a Juggernaut Car, with twin deity of medicine and surgery, driven by pathology. On closer examination, however, it will be found that many of the prescribed courses are short, and can be attended concurrently. It will probably be found that the total amount of attendance required on lectures, laboratory and clinical work is about the same as what is insisted on at all the newer Universities.

Amongst the many obvious advantages of a thorough and comprehensive course, it must not be forgotten that it renders numerous examinations for the degree less imperative; but examination for a degree in medicine is obviously far more necessary than in science, and it must not be forgotten that of the five and a half years' study required the student need only spend three in Bristol.

In order to introduce the stimulus of competition, and to afford recognition of specially good work, a scheme is under consideration for granting honours or distinction in the examinations for the M.B. and B.Ch. Further, an arrangement has been made to allow old students of the University College, Bristol, to enter for degrees in medicine and surgery up to May, 1912. It is possible that an examination for old students may be held towards the end of the ensuing summer session.

The arrangements for clinical teaching at the Royal Infirmary and General Hospital have been re-organised, and every day of the week (except Saturdays) one or more physicians and surgeons have undertaken to devote their whole time to definite clinical instruction at the bedside. These "teaching ward rounds" will be continued throughout the year. In addition to this, Clinical Demonstrations in the special and out-patient departments are arranged daily (excepting Saturdays) during term time. It is hoped that these courses of demonstrations may be so co-ordinated that the whole domain of clinical medicine and surgery will be covered.

Another new departure is the appointment of Demonstrators of Anatomy and Physiology to give their whole time to teaching those two subjects.

**Commemoration Service.**

On December 7th a service in commemoration of the founding of the University was held in the Cathedral. The Lord Mayor and Corporation attended the service, and a large number of representatives of the Council, Senate and teaching staff of the University attended. The first University sermon, which we hope will become an annual event, was preached by the Rev. A. A. David, the newly-appointed Head Master of Rugby.

**The University Colston Society.**

The eleventh annual dinner of the University Colston Society was held on February 17th, when Sir William Ramsay, who was the guest of the evening, related various interesting experiences which befell him when he came to University College, Bristol, thirty years ago. He spoke very emphatically on the necessity of adequate payment of professors, lecturers and demonstrators, so that they may be enabled to do the best work they are capable of, so that the pressure of monetary trouble may not interfere with their productiveness. Many of the American colleges have received large gifts. The temptation has too often been yielded to expend these gifts in bricks and mortar, and in increasing the number of the staff, without increasing their remuneration. He also supported the growing opinion that scholarships are mostly a waste of money. They should only be awarded to those whose financial circumstances prevent them obtaining education. Granted as loans, on evidence of power of application and of good conduct, they would in most cases be more profitably bestowed.

**Presentation to Professor Lloyd Morgan.**

On March 2nd many of the friends and admirers of Professor Lloyd Morgan met together to present him with a gift of silver plate and bookcase, together with a large number of scientific works bearing on subjects in which Professor Lloyd Morgan has taken such a deep interest, and also to present Mrs. Lloyd Morgan with a gold bracelet. It is singularly fitting that the recognition of so eminent a leader of thought in the department of science to which Professor Lloyd Morgan has more particularly devoted himself should be made thus early in the life history of Bristol University, for inasmuch as the Professor had for many years, as Principal of University College, led and guided that Institution towards its culmination in the Bristol University, it not rarely happens that in his native town or in the city of his adoption even a very distinguished man of letters or science, who is fittingly recognised in literary and scientific circles throughout the world, is not adequately appreciated by those amongst whom he dwells. Indeed, it is probable that the report of the proceedings at the University of Bristol may have conveyed for the first time to many citizens of Bristol the information that in the well-known figure of Professor Lloyd Morgan we had one amongst us whose name is honoured among scientists in all quarters of the globe. The very high estimation in which Professor Lloyd Morgan is held by those who have been associated with University College was well expressed in the opening remarks by the Right Hon. Lewis Fry, who presided over the meeting. He pointed out that amongst those two hundred and thirty friends and admirers who had taken part in the scheme were included many members of the Council and Staff of University College, and a large number of past and present students, and of other friends in Bristol and various parts of the country. By their presentation, it was their desire to mark their great sense of appreciation of Professor Lloyd Morgan's services, not alone in leading and developing Bristol University College, but also his services in the cause of higher education, and his marked influence in raising and maintaining the standard of intellectual and social life in Bristol.

Professor Lloyd Morgan expressed his gratification at the presentation which had been made to his wife, and his sincere thanks for the beautiful gifts to himself, and went on to say that he felt that he had been listening to his own obituary, for as Principal of University College he was dead, but he hoped he should have some few more years of life to serve the University.

He told his assembled friends that he was brought up at a Grammar School at which Classics took a very prominent place, and then proceeded to a College of Chemistry and School of Mines, where he studied under Professor Tyndall and others and was trained to be a Mining Engineer. He finished his training and went to America, where in the course of wide travel in North and South America the one thing specially impressed on his mind was the large amount of ignorance that still remained with him. He therefore took another two years of study under Professor Huxley, and to that scientist he owed a great debt of gratitude. He later received an appointment at the Cape, whence he came to Bristol. When Sir William Ramsay left he accepted the office of Principal with considerable diffidence, and though he undertook it with the idea of filling it temporarily, the period had lengthened out to twenty-two years.

**The Medical Profession and the Reports of the Poor Law Commissioners.**

SURROUNDED as we are with questions of the deepest scientific interest, and realising the truth of the words of the father of medicine, that life is short but art is long, and opportunity fleeting and judgment difficult, it is hardly to be wondered if we sometimes neglect matters which are vitally important to our well-being, and which profoundly concern our status as medical practitioners.

With the object of arousing our attention to the recommendations embodied in the Reports of the Poor Law Commission, and with the hope of stimulating our enquiries into the effects which are likely to follow if such proposals become law, Dr. J. Smith Whitaker gave an interesting and suggestive address on the subject on January 13th in the Medical Library.

> "There is a tide in the affairs of men
> Which, taken at the flood, leads on to fortune;
> Omitted, all the voyage of their life
> Is bound in shallows and in miseries."

However sceptical we, as a profession, may be as to the truth of the first part of this statement, there is little doubt in the minds of those who have carefully studied the Reports that if some, at least, of the proposals are carried into effect the voyage of the life of many of us will certainly, in the end, be bound in shallows and in miseries, one seeing danger looming in one proposal, and another in another, and each estimating the extent of the trouble according to his individual experience and idiosyncrasy. Obviously, then, it is a duty which we owe to ourselves to pay some serious and impartial attention to matters which so intimately affect us, in order that we may have confidence in our opinions, and so feel able to offer to our legislators advice which may have some influence in the maturing of their plans.

It is conceded on all hands that some reforms are necessary, and it is generally agreed that when the political horizon is clearer, and the " somewhat varied and exciting parliamentary possibilities of the present situation " have blown over, the matter will be seriously taken up by one Government or another, and alterations more or less drastic made in our laws. Both Majority and Minority Reports recommend far-reaching changes, and to both serious objections may be raised.

While ready to concede to all the Commissioners the highest motives and the sincere desire to act for the best interests of the community, we cannot help feeling that our profession was inadequately represented on the Commission, and that the opinions of those of us who have had life-long experiences in the working of Poor Law Medical Relief and Contract Practice were not sufficiently consulted.

Broadly speaking, we may say that the chief characteristic of the Majority Report is the attempt to develop individual responsibility by the encouragement of Provident Dispensaries, while the Minority aims more especially at stimulating the consciousness of social obligations. Both aims are excellent in themselves, but, like all virtues, liable to abuse if too self-assertive and wanting in the due consideration of the claims of others. The members of our profession are anxious to look at the question from as many points of view as possible, and willing to subordinate their own interests to the good of the State, but at the same time they are not anxious to be over exploited or wholly immolated on the public altars. Our lives are even now not passed altogether on beds of roses. At times, in weak moments, the spirit of Ulysses fails us, and, like the Lotus Eaters, we feel that

> "We have had enough of action, and of motion we,
> Roll'd to starboard, roll'd to larboard, when the surge was seething free."

If the condition of our lives becomes still harder, if our sons realise that after a preliminary training of ever-increasing pressure the exigencies of practice demand more and more onerous and exacting duties, they will seek other fields for their energies, and refuse to enter our profession. The progress of the science and art of medicine and surgery will be hampered, and the chief aim of the Commissioners, the diminution of poverty—which, by their own showing, is largely due to disease and ill-health—will be defeated.

According to the Majority Report, the Poor Law Medical Officer is in time to be abolished, and the poor allowed to choose their own doctor, or one of several attached to a dispensary. In a paper recently read before the Cambridge and Hunts Branch of the British Medical Association, Dr. Joseph Griffiths says, "It is time for us to wake up, else the State will make us contribute not only our share of the rates and taxes, but also a good portion of our knowledge and skill towards the treatment of the sick poor. We shall have thrown upon us not only the burden of the citizen, but also an additional burden in the shape of charity—indeed, we shall be slaves to the community, and shall be regarded as men whose duty it is to pay all rates and taxes, and besides attend the sick poor on a provident basis. . . . This is nothing less than an imposition, and, once put on, will hang round our necks for many generations."

Equally severe strictures may be laid on the Minority Report. In the opinion of many the relief given under this scheme must in time become gratuitous in the vast majority of cases, and as it is to be given to the individual not only during a condition of destitution, but before destitution arises and subsequently, it will not only increase the number of those legally entitled to relief, but will greatly lengthen the period of relief given. The present expenditure upon the Poor Law in England alone is £15,000,000 a year. Under this scheme it would be enormously increased, and the excessive taxation required would tend to intensify the miseries of unemployment. Besides, such a huge eleemosynary system could not be established over the country without impairing the instincts of thrift and independence. It would have the effect of splitting up the family, and impairing those obligations which are already being weakened, but which ought always to be encouraged and cherished as the very foundation and anchor of a happy and well-ordered state. And it would result in such an encroachment on the liberties of the individual which would never be tolerated, and in a system of surveillance and inquisition from the cradle to the grave.

This naturally leads us to the thought whether modifications and improvements of the present system may not be preferable to revolutionary methods. We must not shut our eyes to the fact that the Poor Law has shown constant advances and improvements, especially since 1834. The infirmary system has been introduced, and, with regard to classification, although there have no doubt been many shortcomings, much has been done to take away the stigma. Thus, as Dr. Downes, one of the Commissioners, shows, of the indoor poor, exclusive of lunatics in asylums and of casuals, more than 38 per cent. were in 1908 already provided for in specialised institutions quite apart from the ordinary workhouse, and the proportion in London was more than 50 per cent. As he says, "There may, indeed, be a danger of classification being carried too far, to the verge of hardship or even tyranny. The breaking up of families, the removal of old folk from their associates and friends, may outweigh many administrative advantages." Again, to take another example, of all children in receipt of relief, not 7 per cent. are housed in a workhouse proper, and there is hardly a Union which does not send this remnant out to the public elementary school to mingle with their fellows. In 1838 nearly half of the entire number of the inmates of the workhouses were children under sixteen years of age. Now only one-tenth of the inmates of workhouses are children under sixteen, and of these one-half are in the sick wards or separate infirmaries, and a large proportion of the remainder are babies under three years of age. Again, within the same period the number of paid nurses of the sick indoor poor has risen from less than 200 to more than 6,000, many of whom are highly trained in their profession.

The beds in the separate infirmaries of London alone now nearly equal, if they do not outnumber, those of all the general hospitals in the whole country. It is a significant proof of the public appreciation of these infirmaries that the proportion of the total deaths in London which occur in the Poor Law Institutions has risen from 12.3 per cent. in 1890 to 20.5 per cent. in 1907. "We should be careful," says Dr. Downes, "in considering statements of cases in which the Poor Law is asserted to be deterrent, not to lose sight of these broad truths."

Revolution is more alien to the mind of our nation than reform, and although it occasionally happens that violent upheavals are the only remedies we can usefully employ, we must never forget how much we owe to those gradual changes which have made our country

"A land of settled government,
A land of just and old renown,
Where Freedom slowly broadens down
From precedent to precedent."

W. A. S.

**Winsley Sanatorium.**

The report for the year 1909, adopted by the annual meeting of subscribers and representatives of corporations and owners of beds held at the Sanatorium on February 26th, is before us. It gives a record of very good work and of increasing utility. It shows a very decided improvement in the selection of suitable cases as compared with former years, but the number of advanced cases received is still too large. Many of these can only make slight temporary improvement, and they occupy beds which should be devoted to earlier and more hopeful cases. We observe that of Class I, the good cases, 84 per cent. were discharged as fit for work, whereas of Class III

only 29 per cent. were fit for work, although even of these some 77 per cent. had much improved during their few months of sanatorium life. The Medical Board is of opinion that it would be most desirable that all cases should be seen by the Medical Officer to the Institution before admission, but this is not possible in the majority of patients scattered throughout three large counties. In the case of patients who can go to the Sanatorium for examination the Medical Officer to the Institution is at all times willing to fill in the second certificate on behalf of the Board of which he is a member.

